# Stakeholders' opinions about a tobacco policy in Lao PDR

**DOI:** 10.1186/1617-9625-5-2

**Published:** 2009-01-14

**Authors:** Tanja Tomson, Kongsap Akkhavong, Hans Gilljam

**Affiliations:** 1 Karolinska Institutet, Department of Public Health Sciences, division of Social Medicine, Sweden; 2 National Institute of Public Health, Vientiane, Lao PDR; 3 Stockholm Centre for Public Health, Tobacco Prevention, Stockholm, Sweden

## Abstract

The global epidemic of tobacco smoking is expected to impact hardest in low- and middle- income countries (LMIC). There is a lack of understanding regarding the policy environments within which tobacco control policies are being introduced particularly in LMIC. This study aims at exploring key stakeholders' beliefs about a tobacco policy in Lao PDR.

This is a qualitative case study with a standardised open-ended questionnaire answered by eleven stakeholders in leading positions within different ministries and the media, donors and NGOs. Themes included the perception of tobacco among professionals, awareness of tobacco as a public health issue, importance of inter-sectoral cooperation, and obstacles faced in implementing policies. The research team included both outsiders and an insider. Analysis was done using the case and cross-case analysis.

Among the respondents there was consensus regarding the positive impact of a national tobacco policy with the exception of the representative from the Ministry of Agriculture. Stakeholders identified education, awareness creation through media and law enforcement as important interventions, followed by taxation. Education should be diversified in the way it should be delivered. It was emphasized that people in rural areas and minority groups need tailored made approaches. A major limiting factor in moving tobacco control forward in LMIC was stated to be the lack of funding. The refusal by tobacco industry to participate in the study is noteworthy. It is essential to draft a national tobacco policy that can help the government to increase taxes, and create adequate provisions for the enforcement of tobacco laws and regulations.

## Introduction

Although tobacco use has declined in many high-income countries, recent decades show sharp rises in tobacco use, especially among men, in low- and middle-income countries (LMIC) [1]. This has fuelled by falling real prices and rising incomes that have made cigarettes increasingly affordable, as well as by aggressive and sophisticated tobacco advertising. By 2030, more than 80% of tobacco deaths will be in LMIC [1]. Close to 60% of the 5,700 billion cigarettes smoked each year and 75% of tobacco users are found in LMIC [2]. Countries and development agencies are increasingly recognizing that tobacco use has negative implications for development that go beyond damage to health outcomes, life expectancy and people exposed to second-hand smoke. Yet, the tobacco industry continues its global expansion, with consumers in LMIC being especially susceptible to its marketing tactics [3].

In Lao People's Democratic Republic (Lao PDR) one of the poorest countries in the world with an overall smoking prevalence of 35% [1] a tobacco decree was approved by the Ministry of Health (MoH) 2001. Recently the MoH approved a regulation calling for health warnings to be printed on cigarette packs and cartons (No 661/MoH/2006) [4] and a regulation on the establishment of smoke-free areas at the National University of Lao PDR (No 3174/NUOL/06) [5].

While extensive scientific evidence exists on the tobacco epidemic, a lack of understanding is still found in policy environments within which tobacco control policies are being introduced and implemented. This is particularly pronounced in LMIC, where the global epidemic of tobacco smoking is expected to impact hardest and control policies face the greatest political challenge [6].

Policy studies to explore responses of stakeholders across areas of tobacco control policy in Thailand and Zimbabwe revealed that there is an urgent need to expand the number of policy studies in low-income and middle-income studies [6]. This study aims to explore key stakeholders' beliefs about a national tobacco policy in Lao PDR.

## Methods

This was a qualitative case study [7,8]. Case studies are in-depth investigations of a single instance of a phenomenon in its real-life context [9]. Stakeholders' beliefs about tobacco as a health problem a future tobacco policy constituted the case. We used a standardised open-ended questionnaire assessing important information regarding the national tobacco policy. The study population comprised of stakeholders e.g. officials in executive positions or leading positions in Vientiane, Lao PDR. In this study it is argued that the involvement of stakeholders increases their likelihood of acting upon the findings from the research [7,8]. In addition, the involvement of stakeholders enables a plurality of perspectives to be obtained and increases the range and quantity of information available to the researcher [8,10]. Due to time limitations only stakeholders holding senior positions were interviewed [11]. This consideration, along with the potentially vast experience that the individual stakeholders may have had regarding a tobacco policy, resulted in an approach that adhered to what Patton [7,8] referred to as a philosophy of breadth rather than depth in order to include the views of all the key stakeholders'.

### Sample

A purposive sample was made which focused upon the researchers' conscious selection of certain subjects to be included in the study [8]. This was done to ensure a broad spectrum of representatives. Those were: Ministry of Health, (MoH), Dept. of Health Information and Education for Health (Dept. HIEH); Department of Hygiene (Dept. Hyg.); Mahosot Hospital (a tertiary hospital in the capital); National Institute of Public Health (NIPH); Ministry of Education, drug unit (MoE); Ministry of Commerce (MoC); Ministry of Agriculture (MoA); Ministry of Information and Culture (MoIC), Dept. of Mass Media; as well as from organisations such as Lao Women's Union; Adventist Development and Relief Agency (ADRA) a non-government organisation; the Association of Southeast Asian Nations (ASEAN) and the tobacco industry. The WHO regional office in Vientiane sent 12 requests to all the selected organisations, and personal appointments were made with the directors/head of department where possible. On two occasions, the directors were not available and individuals in other positions were interviewed instead. The study team comprised both outsiders and an insider [12]. Being outsider (TT, HG) enables curiosity with the unfamiliar and being seen as non-aligned with stakeholders. The insider with major experience from Lao public health and health system meant easy access and authentic understanding of the culture. Both outsiders were experts in global tobacco policy issues.

### Data collection and analysis

The questionnaire was pre-tested in a pilot study. Data collection was carried out between 15–19 September 2003. Interviews were conducted by the first author and in eight cases with the assistance of a translator from the Centre of Health Information and Education. The interviews took approximately one hour with notes being taken. Taking the respondents' through the same questions, with the inclusion of probing questions, ensured that the interview time was used to the fullest. Policy analysis was in the frame of context, content and actors with focus on the latter [13].

Data were thematically coded and categorised according to relevant themes. All data were analysed at the Stockholm Centre for Public Health, Sweden. The questionnaire consisted of eight questions: 1) *How do you as a professional perceive the tobacco issue? *2) *How do you look upon tobacco as a public health issue in Lao PDR? *3) *Do you think there is a need of any kind of intervention in relation to tobacco to protect public health in Lao PDR. If yes, why? *4) *What kind of interventions? Name up to five most important tobacco control measures (rank them) *5) *Who are responsible for the respective kind of interventions? *6) *Which if any are priority groups for these interventions? *7) *Your own organisations role if any in Lao tobacco control *8) *Is there any budget for tobacco control, if yes how much is spent/yearly?*

The analysis stage used what Patton [7,8] referred to as case and cross-case analysis. This approach meant that each interview was considered as a separate case and then compared to other cases to ascertain variations in answers.

Ethical clearance was obtained from the Ministry of Health in Lao PDR and verbal consent was obtained from each subject.

## Results

In total, 11 stakeholder interviews were conducted. The tobacco industry in this case Lao Tobacco Limited (LTL) refused to participate by not responding to the WHO request letter. A second follow-up letter was sent but no answer was received.

Several themes emerged from the analysis of data that related to the potential impact of a tobacco policy. These included the perception of tobacco as a professional, awareness of tobacco as a public health issue, importance of inter-sectoral cooperation and obstacles faced by policy makers (Table 1). General consensus was found among the different stakeholder categories with two exceptions regarding the positive impact a national tobacco policy entails. This pattern was true for all themes that emerged (Table 1). However, the positive opinion regarding a tobacco policy was generally identified by stakeholders' with a public health perspective. The others were the Ministry of Commerce and Ministry of Agriculture (Table 2).

**Table 1:** The coding framework with themes

**1. Awareness of tobacco as a public health issue**	
a. Health hazards	
b. Financial	
c. Policy	
**2. Perception of tobacco as a professional**	
a. Positive	
b. Negative	
**3. Importance of inter-sectoral cooperation**	
a. Ministry involvement	
b. Example of interventions	
c. Priority setting	
**4. Obstacles**	
a. Economical	
b. Sustainability	

**Table 2:** Perceptions of tobacco as public health issue and a future national tobacco policy (+ in support of, - against, +/- ambivalent)

**Stakeholder**	**Tobacco public health issue**	**Tobacco policy**
Ministry of Health	+	+
Dept Health Information and Education for Health	+	+
Dept of Hygiene	+	+
Mahosot hospital	+	+
National Institute of Public Health	+	+
Ministry of Education	+	+
Ministry of Commerce	+/-	+/-
Ministry of Agriculture	-	-
Ministry of Information and Culture	+	+
Lao Women's Union	+	+
ADRA	+	+
ASEAN	+	+
Tobacco Industri	n.a.	n.a.*

*n.a.- not applicable

### Awareness of tobacco as a public health issue including the need of a policy

All respondents elaborated upon the tobacco as a public health issue except one. The dissenting opinion about tobacco as a public health issue was expressed:

"I don't see any public health issue here in Lao. If we don't grow tobacco the poverty will increase. ...But everybody knows the harmful effects of smoking already."

(MoA)

Two stakeholders representing the view of the absolute majority gave an example of tobacco as a public health issue said:

"Life expectancy is relatively low, about 55 years. When I came here 8 years ago it was 43 so in ten years it will probably be 65. Then tobacco will be a real public health issue for the people here in Lao PDR."

(ADRA)

"I see more of cardiovascular diseases, lung cancer, bronchial tumours and hypertension. It is important to educate all our staff about the side effects from tobacco.... Smoking in public areas is a huge problem."

(Mahosot hospital)

Additionally, another stakeholder commented that:

"*In general we have low incomes in Lao, one has to decide what to buy – food or cigarettes. Both is not possible you have to choose."*

(MoC)

All but one of the respondents perceived a tobacco policy as positive and referred to it as follows:

"People from various ministries: customs, education, police, finance, information and culture, communication, industry must try to expand the tobacco policy to National Tobacco Control Policy."

(ASEAN)

"Tobacco policy exists but no regulation. For example, we lack of smoke-free areas indoors."

(MoI&C)

The policy doesn't allow smoking but they smoke in front of teachers." ...a Policy of tobacco control is approved officially."

(Dept. HIEH)

The respondent from Ministry of Agriculture was negative:

**"***My organisation is responsible for crop development, to promote it as an industry and to eliminate the poverty alleviation."*

Whereas the Ministry of Commerce was more ambivalent:

***"****We are only giving licences for selling and buying. The Ministry of Agriculture and the industry (tobacco) are those responsible for this product... The Ministry of Commerce try to increase the tax for the cigarettes"*.

In Table 2 the stakeholders' perceptions of tobacco as public health issue and a future tobacco policy are presented.

### Perception of tobacco as a professional

All the respondents except one identified tobacco and smoking as a public health problem. Particular mention was given to the problem among minority groups in the rural areas. An example given was:

"The tobacco issue is a problem everywhere, mostly I see it among the minorities who live in the remote and mountainous areas." (Lao Women's Union)

Another referred to it as an increasing problem and a future challenge:

"It is an interesting challenge. People who smoke have limited access to mass information."

(ADRA)

One respondent commented that:

"Tobacco crop cultivation is an industry to supply the demand, it's good for agriculture ....this crop eliminates the poverty."

(MoA)

### Importance of inter-sectoral cooperation

Several of the respondents, particularly those within various ministries, referred to cooperation within and outside ministries as an imperative, as illustrated in three statements:

"Co-operation with other ministries is important."

(MoI&C)

"It is very important to strengthen the collaboration between sectors -central, district and provincial."

(NIPH)

All stakeholders' except one identified the most important intervention as education. They diversified the way it should be delivered and emphasized that people in rural areas and minority groups are illiterate and other approaches than the common one must be utilised. Further, awareness creation through media for the young was mentioned as a priority area. The second most important intervention indicated was law enforcement and the third taxation. Other opinions of different stakeholders' included:

"Health education, advice to key persons, monks, teachers who teach adults, not only school children."

(Dept. HIEH)

"Education in different ways. Through radio, or a dialog on TV for example, every year near the smoke-free day you could use of one patient who had smoked for a long time and another one who succeeds to quit."

(Dept. Hyg.)

"...we have to do a lot more work to educate, and inform about the harm tobacco can cause to health."

(ASEAN)

"Second, the law regulation, third is the taxation. We should have high taxation, then, this revenue should be used to do research for promoting health..."

(NIPH)

Another stakeholder expressed his view about interventions and priority setting as follows:

"We should start with awareness creation and target young people. In some ethnic groups, tobacco is used as a relaxant. Mothers who carry their children on their back used to smoke and while doing it exhale the smoke on the child's face (believing it calms the children)."

(ADRA)

### Obstacles

All respondents except one mentioned the financial issue as one of the most important concerns. Funding from international sources is scarce as are earmarked funds within the Ministry of Health and other ministries. Sustainability was another obstacle which was related to the former. This is illustrated by three of the stakeholders below:

"We work by ourselves, it's difficult to achieve success. TV and media have wanted to co-operate. The national radio wanted to broadcast but since we don't have any money, nothing happened. Funding is an important issue."

(Dept. HIEH)

"Funding is difficult. We received some money from Australia-Education Strategies. It is not enough we need much more to be able to have people who work with the tobacco issue continuously."

(MoE)

"There is a vacuum in this sector. No direct interventions exist only partial ones. We cannot expect any budget from MoH so there is a funding problem, we have to find out how to raise funds and to do it in a multi-disciplinary approach. Some NGOs have given some funds but nothing has been done at a national level, only in some regions."

(ASEAN)

One of the stakeholders had another approach to funding and stated:

"Here in Lao only tobacco has high technology processing. For sugar cane, cotton, soya beans we don't have it. We would like to have a sugar cane factory. Can you get the money for us?"

(MoA)

## Discussion

Most stakeholders' perceived the idea of a national tobacco control policy positively and had good awareness of public health concerns. Using Walt and Gilson's framework, this study has focused on the actors. Usually actors are neglected, and policy analysis often wrongly focuses its attention on the content. Thus, we found it appropriate to emphasize the stakeholder perspective [13].

There was actually high awareness of the health consequences among stakeholders' in contrast to what was reported in another study from Hong Kong [14]. In particular, all the respondents except one were clear about the fact that tobacco is a public health issue, that there is a need for tobacco control interventions, and about the types of interventions needed. This may be due to the extensive tobacco control efforts that have been underway in Lao PDR, and to the fact that globalisation and the intervention of external actors such as Thailand with 30 years of experience in tobacco control may have increased [6].

Most of the stakeholders' emphasized that enforcement of laws banning smoking in public areas is important, and a development of a tobacco policy decree to a national tobacco policy is required. This is under development, and MoH plans to revise the tobacco policy into a national tobacco law (personal communication WHO/TFI). It is also known that successful interventions will require many stakeholder groups to take action at the local, national, and international levels [6].

The stakeholders' representing Ministry of Commerce (being ambivalent towards a national tobacco policy) and Ministry of Agriculture (negative towards a national tobacco policy) don't have the same agendas as the rest of the representatives which may be justified by their involvement in tobacco cultivation.

Inclusion of opposing views is a prerequisite for understanding the political environment and for finding opportunities for change [15]. This may help in the planning of strategies to overcome their opposing ideas. However, the overwhelming consensus among the actors included gives us a good opportunity to understand how they think and helps us assess strengths and weaknesses on this specific issue.

The refusal by one of the stakeholders to participate is likely due to the matter in question. While other industries use corporate social responsibility to address social issues, the tobacco industry protects their core business product. As such, the corporate nature of tobacco companies is a structural obstacle to reducing harm caused by tobacco use [16]. Therefore, the expressed defensiveness might be a natural way to behave for those with a vested interest.

As is shown also in this study, a major limiting factor in moving tobacco control forward in LMIC is the lack of funding. Government support and financial commitment to tobacco control vies with other pressing health and economic concerns in developing countries like Lao PDR [17].

However, the remaining challenge is to give much greater priority to tobacco control policies and to convince international organisations and donor agencies that 0,05% of all financial aid should be earmarked for tobacco control.

The purposive sampling to cover the full range of possible opinions and experiences, rather than random sampling to produce a representative study group was a prerequisite. The interviewer (TT) was an outsider with no relationship to the subjects but some bias may have been introduced such as data affected by characteristics of the interviewer as well as by the interactions of the interviewer/respondent. Respondents may have felt that their answers were not going to remain anonymous and were less open than otherwise was possible. They may think they should answer in a certain way because they thought they were being checked [18]. Translation is another bias but the literature suggests that few translation errors occur when the translators, as was the case in this current study, are able to adequately relate to the content of the source of language [19]. The fact that the research team combined insider and outsiders perspectives during data collection, analysis and interpretation enabled a more comprehensive understanding.

It may be argued that these findings cannot be generalized beyond the present study, but in fact the use of a theoretical sampling frame greatly increases the transferability of the results that of a "convenience" sample [20].

We show that stakeholder analysis is a tool that can help explore views of key persons, which in turn helps policy makers to become aware of forces operating for and against tobacco control in their country.

By focusing on the actors' perspective in Lao PDR, the study was able to highlight the beliefs, and attitudes of key stakeholders playing an important role for implementation of tobacco control agendas and finally a national tobacco policy.

The key messages indicate that it is essential to draft a national tobacco policy that can help the government to increase taxes, and create adequate provisions for the enforcement of tobacco laws and regulations.

## Competing interests

The authors declare that they have no competing interests.

## Authors' contributions

TT conceived the idea for the study. TT and KA developed the questionnaire and TT conducted the interviews. TT wrote the fist draft, everybody commented and all authors read and approved the final version of the manuscript.
